# SYN023, a Humanized Monoclonal Antibody Cocktail, Broadly Neutralizes Genetically Diverse Lyssaviruses and Validates Epitope Prediction Accuracy

**DOI:** 10.3390/vaccines14100862

**Published:** 2026-09-29

**Authors:** Jiagui Qu, Charles E. Rupprecht, Pengcheng Yu, Wuyang Zhu, Shiyun He, Qun Lin, Eric Tsao

**Affiliations:** 1 Synermore Biologics (Suzhou) Co., Ltd., Suzhou 215000, China; jgqu@synermore.cn (J.Q.); syhe@synermore.cn (S.H.); qunlin@synermore.cn (Q.L.); 2 College of Forestry, Wildlife and Environment, Auburn University, Auburn, AL 36849, USA; charleserupprechtii@gmail.com; 3 College of Veterinary Medicine, Auburn University, Auburn, AL 36849, USA; 4 NHC Key Laboratory of Medical Virology and Viral Diseases, National Institute for Viral Disease Control and Prevention, Chinese Center for Disease Control and Prevention, Beijing 102206, China; yupc@ivdc.chinacdc.cn (P.Y.); zhuwuyang1971@sina.com (W.Z.)

**Keywords:** SYN023, lyssavirus, pseudovirus system, epitope prediction, broad-spectrum neutralization, rabies, humanized monoclonal antibody cocktail

## Abstract

**Background/Objectives:** Lyssaviruses pose a persistent global public health threat, underscoring the need for broad-spectrum neutralizing antibodies to improve rabies post-exposure prophylaxis (PEP). The humanized monoclonal antibody cocktail SYN023 is approved for PEP in China and under active global regulatory review. This study aimed to validate pseudovirus-based neutralization assays, verify SYN023 epitope prediction accuracy, and systematically characterize its pan-lyssavirus neutralizing activity. **Methods:** Assay validation was performed by directly comparing neutralization titers obtained with authentic rabies virus (RABV) and pseudotyped virus. Epitope analysis was performed, followed by in vivo evaluation in a canine PEP challenge model. The pseudovirus-based neutralization assay was used to assess SYN023 broad-spectrum activity across diverse lyssaviruses. **Results:** Neutralization titers from authentic and pseudotyped viruses showed high concordance (R^2^ = 0.8341, *p* < 0.0001), supporting the pseudovirus system as a reliable surrogate assay. Epitope analysis confirmed that SYN023’s epitope is fully conserved in the highly virulent New York City (NYC) RABV strain. In canine PEP challenge studies using the NYC strain, SYN023 conferred strong protection at 0.1–0.5 mg/kg. SYN023 exhibited potent neutralizing activity against phylogenetically diverse non-rabies lyssaviruses, including Mokola virus (MOKV; EC_50_ = 14.74 µg/mL), whereas three clinically validated reference antibodies failed to neutralize MOKV even at concentrations up to 1000 µg/mL. **Conclusions:** This study demonstrates that the pseudovirus-based neutralization assay is a reliable surrogate assay. It also confirms the functional accuracy of the epitope mapping strategy and demonstrates the broad neutralization spectrum of SYN023 against a diverse panel of lyssaviruses.

## 1. Introduction

The *Lyssavirus* genus, within the family Rhabdoviridae, comprises RABV and 18 other phylogenetically distinct non-RABV species [1]. All lyssaviruses are zoonotic pathogens capable of causing acute, progressive, and invariably fatal encephalitis that is clinically indistinguishable from classical rabies [2]. Beyond RABV, the spillover of non-RABV lyssaviruses into humans has resulted in fatal clinical cases across Africa, Europe, and Australia, representing an under-recognized public-health risk. In South Africa, two fatal cases caused by DUVV were reported in 1970 and 2006 [3,4]. In Europe, five human deaths associated with European bat lyssaviruses 1 and 2 (EBLV-1, EBLV-2) have been recorded since the 1980s [5,6,7,8]; one fatal case involved an occupationally exposed patient, where routine rabies diagnostics returned false-negative results and pathogen identification was only achieved post-mortem by next-generation sequencing [8]. In Australia, four fatal infections caused by ABLV have been reported, including a 2025 case in which a middle-aged male developed fatal neurologic disease months after receiving standard rabies PEP following exposure to a flying fox—providing compelling evidence of incomplete cross-neutralization by currently licensed rabies biologics against antigenically divergent lyssaviruses [9,10,11,12]. MOKV—the sole lyssavirus not associated with chiropteran reservoirs—was responsible for two fatal encephalitis cases in Nigeria during the 1970s following contact with small mammals [13].

Globally, lyssavirus infections account for tens of thousands of human deaths annually [14]. Although licensed rabies vaccines and PEP regimens are widely deployed, the expanding taxonomic and geographic diversity of non-RABV lyssaviruses, recurrent spillover events resulting in human mortality, and well-documented limitations of existing neutralizing biologics, including narrow spectrum and strain-dependent efficacy, highlight an urgent unmet need for next-generation, broad-spectrum prevention and control strategies. SYN023 is a novel humanized monoclonal antibody cocktail comprising two non-competing antibodies that jointly target spatially distinct, highly conserved conformational epitopes on the lyssavirus glycoprotein [15]. It has demonstrated potent in vitro neutralizing activity against RABV strains isolated from carnivorous mammals worldwide and from bats across the Americas, as well as against four phylogenetically distinct non-RABV lyssaviruses [15,16]. Approved for clinical use in China, SYN023 has been extensively administered as part of rabies PEP in real-world settings, with observational data demonstrating 100% protection against World Health Organization (WHO) category III exposures [17,18].

However, key knowledge gaps persist regarding (i) the breadth of SYN023’s neutralizing activity against phylogenetically diverse non-RABV lyssaviruses; (ii) the regulatory acceptability and analytical rigor of the pseudovirus-based neutralization assay used for potency assessment; and (iii) experimental validation of the epitope predictions underpinning its rational design. To address these interrelated questions and generate mechanism-informed, regulatory-grade evidence supporting global implementation, we performed an integrated study comprising (i) head-to-head comparison of the pseudovirus-based neutralization assay with the authentic virus-based neutralization assay; (ii) functional confirmation of predicted epitopes through in vivo PEP studies; and (iii) systematic cross-neutralization profiling across a representative panel of lyssaviruses, with quantitative EC_50_ determination and benchmarking against commercially available monoclonal antibodies. Collectively, these data fill critical knowledge gaps and provide key support for SYN023’s global regulatory submissions.

## 2. Materials and Methods

### 2.1. Correlation Analysis Between Authentic and Pseudotyped Virus

To evaluate the correlation between SYN023-mediated neutralization of authentic lyssaviruses and pseudotyped lyssaviruses, a total of 22 phylogenetically and geographically diverse lyssavirus variants was subjected to head-to-head comparative neutralization testing using both authentic virus-based and pseudovirus-based assays.

Neutralization testing using authentic virus was performed independently by the Canadian Food Inspection Agency (CFIA), the U.S. Centers for Disease Control and Prevention (CDC), the Chinese Center for Disease Control and Prevention (China CDC), and Kansas State University. Of the 22 variants, neutralization data for 14, including 2 laboratory-adapted strains (CVS-11 and ERA), 5 isolates from carnivorous mammals, and 7 bat-derived isolates, have been previously reported; all exhibited complete neutralization by SYN023 [15,16]. For the remaining 8 variants, 3 (2 carnivore-derived and 1 bat-derived isolate) were evaluated by CFIA and CDC using methodologies published previously [16]; the remaining 5, exclusively isolated from carnivorous mammals, were tested at China CDC. The China CDC neutralization assay protocol is described below: Six 5-fold serial dilutions of SYN023 (1.0, 0.2, 0.04, 0.008, 0.0016 and 0.00032 µg/mL) were pre-incubated with RABV variants in a 96-well plate for 60 min at 37 °C. Subsequently, 100 µL of mouse neuroblastoma (MNA) cells (5 × 10^5^ cells/mL) were added to each well containing the antibody–virus mixture (100 µL SYN023 + 100 µL virus [50 FFU_50_]), with eight replicates per dilution, followed by incubation for 48 h at 37 °C under 5% CO_2_. Cells were then washed, fixed with cold 80% acetone, and stained with fluorescein isothiocyanate (FITC)-labeled anti-RABV immunoglobulin (Fujirebio Diagnostics, Inc., Malvern, PA, USA), supplemented with 0.5% Evans blue in PBS to suppress nonspecific fluorescence. Virus-infected foci were enumerated using a fluorescence microscope at ×200 magnification. The RABV neutralizing antibody (RVNA) titers were calculated from the proportion of positive wells per dilution using the Reed–Muench method and converted to EC_50_ for comparison analysis.

Neutralization testing using the pseudovirus system was conducted as follows: Pseudotyped lyssaviruses were generated by co-transfecting HEK293T cells with a plasmid encoding the lyssavirus glycoprotein (pCAGGS-G) to mediate pseudotyping, followed by infection with VSVΔG*-G (a glycoprotein-complemented vesicular stomatitis virus bearing a luciferase reporter and lacking its native G gene). Prior to pseudovirus infection, HEK293T cells were seeded in 96-well plates and incubated at 37 °C under 5% CO_2_ for 4–6 h to achieve optimal confluence. Serial dilutions of SYN023, clinically referenced monoclonal antibodies (including Rabishield, Ormutivimab and GR1801), or human rabies immune globulin (HRIG; Nanyue Biopharmaceutical Co., Ltd., Hengyang, China) were prepared in assay medium. Each antibody dilution (50 µL) was pre-incubated with RABV pseudovirus (100 µL; 2000–4000 TCID_50_) for 1 h at 37 °C. The antibody–pseudovirus mixture was then added to wells containing the pre-seeded HEK293T cells. Plates were shaken gently to synchronize infection, followed by incubation at 37 °C under 5% CO_2_ for 24 h. At 24 h post-infection, 200 µL of culture supernatant was aspirated carefully from each well, and 50 µL of luciferase detection reagent (Vazyme, Nanjing, China) was added. After brief mixing, luminescence was quantified using a microplate luminometer. Neutralization activity was calculated as percent inhibition of luciferase signal relative to pseudovirus-only controls, and EC_50_ values were determined using nonlinear regression fitted to a four-parameter logistic model.

### 2.2. Experimental Animals

A total of 33 beagle dogs, balanced for sex and approximately 3 months of age, were enrolled in the NYC strain of rabies virus challenge dose titration and SYN023 PEP efficacy studies. Animals were sourced from Covance Research Products (Cumberland, VA, USA) and maintained under a standardized routine vaccination program against canine parvovirus (CPV), canine distemper virus (CDV), canine adenovirus (CAV), canine parainfluenza virus (CPIV), and canine coronavirus (CCV), among other endemic pathogens. Prior to study initiation, all dogs underwent serological screening on day –14 (±2 days) to confirm rabies virus seronegativity; neutralizing antibody titers were below the limit of detection (<0.1 IU/mL) as determined by the rapid fluorescent focus inhibition test (RFFIT). Dogs were individually housed under ABSL-2 conditions with ad libitum access to certified feed and potable water. A 7-day acclimatization period was implemented prior to study procedures to ensure physiological and behavioral stabilization. Any animal exhibiting clinical signs indicative of systemic illness or distress was humanely euthanized in accordance with institutional animal welfare guidelines. Viral challenge, therapeutic intervention, and outcome assessments were conducted in a strictly defined, consistent sequence across all subjects. Cage assignments were randomized and spatially balanced within the housing room to mitigate positional confounding. To ensure blinding, animal care staff responsible for daily clinical observations had no access to treatment group assignments, including both rabies virus challenge dose levels and SYN023 administration schedules, and all related assignment documentation was physically removed from the housing area prior to study commencement. All experimental procedures, clinical monitoring, and data collection were conducted in strict accordance with the SOPs of Labcorp (formerly Covance) to ensure methodological consistency and minimize experimental variability.

### 2.3. Epitope Analysis of the NYC Strain

Samples of highly virulent NYC street RABV (Lot No. 92-5A) were provided by the U.S. Department of Agriculture (https://www.aphis.usda.gov/sites/default/files/cvb-dat-0424.pdf, accessed on 1 April 2026). This live rabies virus was isolated from fox salivary glands and kept at −80 °C. To establish the canine challenge dose, nine dogs were inoculated intramuscularly with three serial dilutions of the NYC strain (1:10, 1:100, and 1:1000; *n* = 3 per group). Rabies virus RNA was extracted from brainstem tissue of four dogs that succumbed to infection. Full-length glycoprotein (G) gene sequences were generated via bidirectional Sanger sequencing: viral RNA was reverse-transcribed and amplified using overlapping RT-PCR; purified amplicons were sequenced with BigDye Terminator v3.1 chemistry on an ABI 3500xl capillary platform. Consensus sequences were assembled from forward and reverse chromatograms to obtain complete G gene coding sequences. Evolutionary conservation of the SYN023 epitope residues—relative to the reference challenge strain CVS-11—was evaluated by multiple sequence alignment using ClustalX 2.1.

### 2.4. PEP Efficacy of SYN023 Against the NYC Strain

Twenty-four beagle dogs were randomly allocated to four treatment groups (*n* = 6 per group) using a computer-generated randomization sequence (Microsoft Excel^®^). Group assignment was stratified by body weight and sex to ensure balanced distribution across all groups. On Day 0, all animals received an intramuscular (IM) challenge with the NYC RABV strain at a 1:100 dilution from the original virus stock ([10^7.9^ MICLD_50_/mL]). Groups 1–3 received a single IM dose of SYN023 at 0.5, 0.3, or 0.1 mg/kg, respectively, administered 24 ± 1 h post-challenge (Day 1) at the same anatomical site as the viral inoculation. The control group (Group 4) received an equivalent volume of PBS administered IM at the inoculation site. Clinical status was monitored daily for 90 days; survival was recorded, and protection rates were calculated as the percentage of animals surviving to the end of the study. Blood samples were collected before treatment and upon onset of clinical signs or at the humane endpoint; terminal brainstem tissue was harvested at euthanasia. Serum was analyzed for RVNA titers using the RFFIT. Brainstem sections were evaluated for RABV antigen detection by direct fluorescent antibody (DFA) test.

### 2.5. RFFIT for Serum RVNA Titer Quantification

The RFFIT assay was performed to quantify RVNA titers in serum samples. Serial five-fold dilutions of each sample were prepared in EMEM-10 medium and loaded onto 8-well chamber slides (Lab-Tek^®^, Waltham, MA, USA). A standard dilution series—1:5, 1:25, 1:125, and 1:625—was achieved by first diluting serum 1:2.5, 1:12.5, 1:62.5, and 1:312.5 in EMEM-10 (final volume per well: 0.1 mL), followed by addition of 0.1 mL of challenge virus (rabies virus CVS-11 strain; 30–50 FFU/mL). Slides were incubated for 90 min at 37 °C in a humidified CO_2_ incubator. Subsequently, 0.2 mL of BHK-21 cell suspension (5 × 10^5^ cells/mL) was added to each well, gently mixed, and incubated for 24 h at 37 °C under 5% CO_2_. Cells were then washed with PBS, fixed in cold 80% acetone, and stained with FITC-conjugated anti-rabies virus immunoglobulin (Fujirebio Diagnostics, Inc., Malvern, PA, USA), supplemented with 0.05% Evans blue (in PBS) to quench nonspecific fluorescence. Slides were examined under a fluorescence microscope at ×200 magnification. For each dilution, 20 microscopic fields were assessed for fluorescent foci. RVNA titers were calculated as the endpoint dilution (ED_50_) using the Reed–Muench method and converted to international units per milliliter (IU/mL) by comparison with the WHO International Standard for Rabies Immunoglobulin (SRIG; assigned potency: 2.0 IU/mL), according to the formula: IU/mL = (ED_50_ reciprocal titer of test sample ÷ ED_50_ reciprocal titer of SRIG) × 2.0 IU/mL.

### 2.6. DFAT for RABV Antigen Test in Brain Tissues

Direct fluorescent antibody testing (DFAT), widely regarded as the gold standard for rabies diagnosis, was employed for qualitative detection of rabies virus (RABV) antigen in fresh brain tissue specimens obtained from experimentally challenged animals. Monolayer impression smears were prepared by gently pressing transverse sections of brainstem tissue onto glass slides, followed by air-drying at room temperature for 15 min and fixation in cold acetone (−20 °C) for 1 h to overnight. Concurrently, positive and negative control slides were processed under identical conditions. After fixation, residual acetone was carefully removed, and slides were allowed to air-dry at room temperature. Subsequently, slides were placed in a humidified chamber and incubated with FITC-labeled anti-rabies virus immunoglobulin (Fujirebio Diagnostics, Inc., Malvern, PA, USA), diluted in PBS containing 4 µL/mL Evans blue dye, at 37 °C for 30 min. The unbound conjugate was removed by gentle rinsing with PBS, followed by two sequential 5 min soaks in PBS. Slides were then vertically drained, air-dried at room temperature, mounted with 20% glycerol in Tris buffer (pH 9.0), and covered with thin glass coverslips. Fluorescence microscopy was performed using an inverted fluorescence microscope at a minimum magnification of 200×. For each brain region, 40 non-overlapping microscopic fields were systematically evaluated by two independent, blinded technicians. A result was considered positive if distinct apple-green, punctate or granular fluorescence was observed specifically within neuronal cytoplasm, in the absence of non-specific red background fluorescence; absence of such specific green fluorescence was interpreted as negative.

### 2.7. Statistical Analysis

Pearson correlation analysis was performed to evaluate the association between SYN023-mediated neutralizing activity against authentic RABV and rabies pseudovirus. Survival curves were compared using the log-rank (Mantel–Cox) test. The RVNA titers were compared between dogs with positive versus negative direct fluorescent antibody (DFA) test results using an unpaired two-tailed Student’s *t*-test. *p* < 0.05 was considered statistically significant. All statistical analyses were conducted using GraphPad Prism version 8.0.

## 3. Results

### 3.1. Reliability of the Pseudovirus System and Accuracy of Epitope Prediction

The correlation analysis between authentic lyssavirus and pseudovirus revealed a strong positive correlation (Pearson R^2^ = 0.8341, *p* < 0.0001), indicating that the EC_50_ values of SYN023 measured in the pseudovirus assay closely mirrored those obtained using the authentic virus system (Figure 1 and Table S1).

As a bispecific antibody cocktail, SYN023 retains neutralizing activity as long as at least one constituent antibody (CTB011 or CTB012) effectively engages its target epitope. Notably, the CTB012 epitope residues are highly conserved across all tested RABV variants (Table S1); correspondingly, SYN023 demonstrated robust and consistent neutralization against these variants in both authentic virus and pseudovirus assays. This concordance supports the utility of epitope conservation as a predictive indicator of cross-neutralization potential. In contrast, the two non-RABV lyssaviruses, BBLV and EBLV2, harbor amino acid substitutions in the epitopes bound by both CTB011 (N355T) and CTB012 (V291I). Nevertheless, SYN023 and CTB012 alone retained measurable neutralizing activity against these divergent viruses, albeit with reduced potency (i.e., higher EC_50_ values; Table S1). Specifically, the V291I substitution within the CTB012 epitope partially attenuated but did not abolish neutralization.

### 3.2. SYN023 Neutralization Prediction and PEP Efficacy Against the NYC Strain

All dogs succumbed to infection following challenge with the NYC rabies virus at dilutions of 1:10 (3/3) and 1:100 (3/3) of the original stock; in contrast, one of three dogs survived challenge at the 1:1000 dilution (Table S2). The 1:100 dilution was selected as the standardized challenge dose, as it represented the lowest viral concentration at which uniform, lethal infection was observed across all animals. Epitope mapping was performed using RNA extracted from brainstem tissue of four dogs that succumbed to NYC rabies virus infection.

Epitope analysis revealed that the NYC strain harbors no amino acid substitutions in either the CTB011 or CTB012 epitope (Figure 2A), indicating full conservation of both target sites and supporting the expectation that SYN023, a cocktail of CTB011 and CTB012, would potently neutralize this strain. Consistent with this prediction, SYN023 exhibited potent neutralizing activity against pseudoviruses bearing the NYC strain glycoprotein (EC_50_ = 7.52 ng/mL). In a lethal post-exposure challenge model using unvaccinated dogs infected with NYC street RABV, SYN023 conferred robust protection across all tested doses (Figure 2B): 100% (6/6) survival at 0.5 mg/kg and 83% (5/6) survival at both 0.3 mg/kg and 0.1 mg/kg. In contrast, all animals in the PBS-treated control group succumbed to infection within 10 days post-challenge. The DFA testing confirmed the absence of RABV antigens in all surviving dogs, whereas all moribund or euthanized animals tested positive (Table S3). Furthermore, the RVNA titers in survivors were significantly lower than those in non-survivors (*p* < 0.0001; Table S3), suggesting effective viral clearance rather than immune-mediated pathology.

### 3.3. Broad-Spectrum Neutralization Efficacy of SYN023 Against Non-RABV Lyssaviruses

The pseudovirus neutralization assay demonstrated that SYN023 exhibits potent and consistent neutralizing activity against all tested non-RABV lyssaviruses, with EC_50_ values of 0.63 ng/mL (EBLV1), 8.221 µg/mL (EBLV2), 7.65 ng/mL (ABLV), 0.96 µg/mL (BBLV), 0.29 µg/mL (TWBLV1), 15.75 µg/mL (TWBLV2), 0.57 ng/mL (GBLV), 53.56 ng/mL (DUVV), 0.54 µg/mL (KHUV), 31.62 µg/mL (ARAV), 14.74 µg/mL (MOKV), 4.84 µg/mL (LBV) and 3.18 µg/mL (SHIBV) (Table 1). In marked contrast, other commercially available monoclonal antibodies, Rabishield [19], Ormutivimab [20], and GR1801 [21], failed to neutralize MOKV even at the highest tested concentration of 1000 µg/mL (Table 2). This complete lack of activity underscores SYN023’s distinctive capacity to neutralize MOKV, a capability not shared by any currently licensed rabies biologics. Collectively, these data establish that SYN023 possesses a uniquely broad lyssavirus-neutralizing spectrum, attributable to its dual-targeting mechanism engaging a highly conserved conformational epitope on the lyssavirus glycoprotein.

## 4. Discussion

Lyssaviruses represent a persistent and severe threat to global public health, underscoring the urgent need for broad-spectrum neutralizing agents to strengthen rabies prevention and control [2,14]. The humanized monoclonal antibody cocktail SYN023 has received marketing authorization in China and is currently under global regulatory review. The present work generated three sets of regulatory-relevant evidence: assay validation for pseudovirus neutralization, functional verification of epitope-based efficacy prediction in a canine PEP model, and systematic profiling of cross-neutralization against a panel of genetically diverse lyssaviruses.

Pseudovirus-based neutralization assays offer substantial biosafety and operational advantages over authentic high-containment lyssavirus work by eliminating the requirement for BSL-3 facilities and enabling scalable, high-throughput serological assessment [22,23,24]. Recent advances have enabled the generation of replication-incompetent pseudotypes representing all 18 known lyssavirus species, greatly expanding the capacity for in vitro antigenic profiling of divergent field isolates [25]. Nevertheless, surrogate assays require empirical validation for each therapeutic candidate rather than direct extrapolation from published platform performance [26,27]. Our data demonstrate a strong correlation between pseudovirus-derived and authentic-virus EC_50_ values (R^2^ = 0.8341, *p* < 0.0001), formally supporting the use of this pseudovirus platform for potency assessment of SYN023. This finding aligns with meta-analytic evidence indicating that, while concordance between pseudotyped and wild-type lyssavirus neutralization readouts varies across antibody candidates, it remains quantitatively informative and mechanistically interpretable [16,28].

Beyond in vitro findings, our canine challenge study using the NYC street-virus strain provided critical in vivo proof-of-concept for epitope-conservation guided-prediction. Full conservation of both CTB011 and CTB012 epitopes in this epidemiologically significant isolate accurately predicted robust protection following post-exposure administration of SYN023. Absence of viral antigen in brain tissue (as confirmed by DFA testing) alongside comparatively low RVNA titers in surviving animals supports a mechanism wherein SYN023 mediates rapid in vivo viral neutralization and clearance, rather than eliciting immune-mediated pathology. These results bridge computational epitope mapping to functional in vivo outcomes and reinforce the utility of glycoprotein epitope conservation as a predictive biomarker for real-world SYN023 performance against circulating field isolates.

A major finding of this study is that SYN023 exhibits exceptional breadth of neutralization against a broad panel of non-RABV lyssaviruses, including representatives from both phylogroup I and phylogroup II, with particularly robust activity against MOKV. In contrast, commercially available rabies immunoglobulin (RIG) and currently approved monoclonal antibody therapeutics show minimal to no neutralizing activity against phylogroup II lyssaviruses, a critical therapeutic gap in light of the documented zoonotic spillover potential of these bat-independent lyssaviruses [19,29]. Fatal human encephalitis caused by MOKV has been historically reported in Africa, and serological surveillance data indicate sustained circulation in small-mammal reservoirs, for which no validated post-exposure prophylactic interventions currently exist [13,29]. Structurally, pan-lyssavirus recognition by therapeutic antibodies is restricted to a limited number of conserved conformational epitopes on the lyssavirus glycoprotein [30]. SYN023 maintains potent activity against MOKV primarily through CTB012-mediated engagement of one such evolutionarily conserved and structurally stable conformational epitope, demonstrating how its dual-antibody cocktail design confers uniquely broad pan-lyssavirus coverage.

Although SYN023 was primarily developed for human rabies PEP, our canine efficacy data support the exploration of potential One-Health-oriented applications [31]. Current guidelines recommend pre-exposure vaccination for domestic animals at risk of lyssavirus exposure [32]. In contrast, unvaccinated companion or livestock animals that experience confirmed or suspected lyssavirus exposure are often subject to euthanasia or prolonged quarantine—measures that impose significant emotional distress on owners and substantial socioeconomic burdens on smallholder farming communities [33]. Field-based programs in regions such as Texas have demonstrated the operational feasibility of administering PEP to unvaccinated exposed animals under rigorously defined local protocols [34]. Passive immunization with neutralizing antibodies represents an underexplored yet biologically plausible alternative for naïve, exposed animals; however, licensed veterinary monoclonal antibody products specifically indicated for lyssavirus prophylaxis remain unavailable [35]. Building on our robust canine challenge data, future studies could systematically evaluate the feasibility, dosing regimen, and clinical utility of SYN023-based PEP in naïve-exposed domestic animals, pending comprehensive assessment of species-specific safety, pharmacokinetics, and cost-effectiveness. Critically, any off-label use in veterinary settings would require formal regulatory review and endorsement by veterinary medical authorities, as well as integration into updated clinical practice guidelines, prior to implementation.

In summary, this study establishes that (i) the pseudovirus assay is a reliable, high-fidelity surrogate for authentic virus neutralization testing of SYN023; (ii) epitope conservation serves as a robust predictor of neutralization susceptibility across diverse lyssaviruses; and (iii) SYN023 exhibits superior, broad-spectrum neutralization against both RABV and non-RABV lyssaviruses. As an already-approved agent in China and undergoing global regulatory review, SYN023 holds substantial promise for expanding the geographic and virologic scope of rabies PEP. The potency data, mechanistic insights, and functional validation reported herein generate robust regulatory-ready evidence supporting its potential integration into national rabies-control programs and WHO guidance [36,37], positioning SYN023 as a promising component for next-generation lyssavirus prevention strategies.

## Figures and Tables

**Figure 1:**
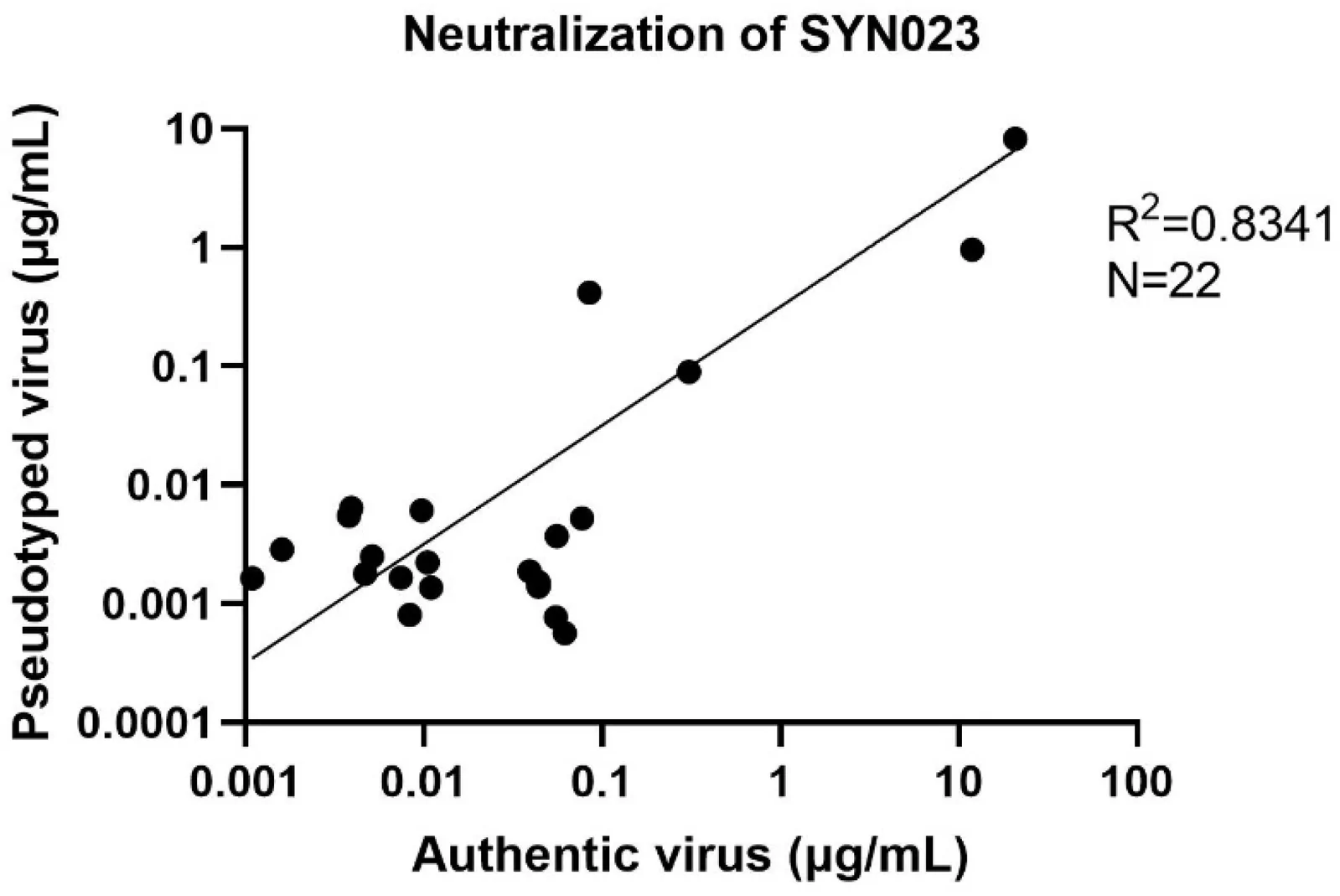
Correlation between authentic virus and pseudovirus neutralization EC_50_ values across 22 phylogenetically and geographically diverse lyssavirus variants.

**Figure 2:**
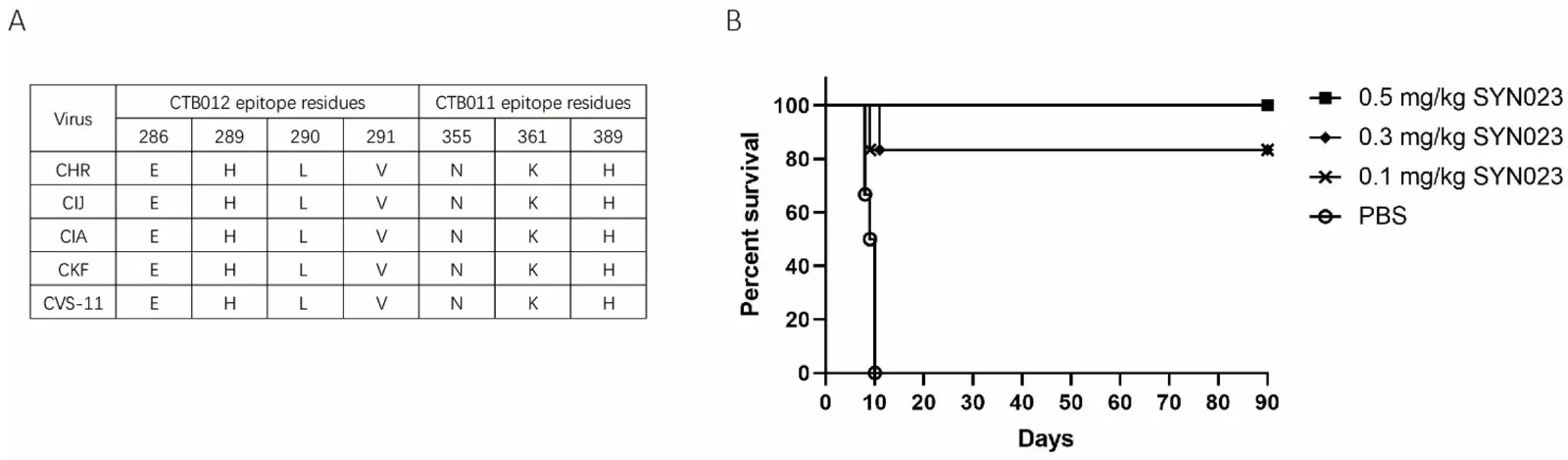
Conservation of the epitope residues in the NYC strain and PEP efficacy of SYN023. (**A**) Glycoprotein sequences of the NYC strain derived from viral RNA isolated from the brainstem of infected dogs (designated CHR, CIJ, CIA, and CKF) were aligned with that of the CVS-11 reference strain. (**B**) Four groups of dogs (*n* = 6 per group) were challenged intramuscularly with the NYC virus and administered a single dose of SYN023 one day post-infection at 0.5, 0.3, or 0.1 mg/kg.

**Table 1:** Neutralization activity of SYN023 against non-RABV lyssavirus variants.

Lyssavirus Variant	GenBank Accession	Lineage	Neutralization of SYN023 (EC_50_)
EBLV1	MF187802	Non-RABV-PI	0.63 ng/mL
EBLV2	JX129233	Non-RABV-PI	8.221 µg/mL
ABLV	AF418014	Non-RABV-PI	7.65 ng/mL
BBLV	MF043188	Non-RABV-PI	0.96 µg/mL
TWBLV1	ON437590	Non-RABV-PI	0.29 µg/mL
TWBLV2	ON437589	Non-RABV-PI	15.75 µg/mL
KBLV	LR994545	Non-RABV-PI	0.33 µg/mL
DBLV	OQ428158	Non-RABV-PI	5.35 µg/mL
GBLV	APD77641	Non-RABV-PI	0.57 ng/mL
DUVV	CAG9056782	Non-RABV-PI	53.56 ng/mL
KHUV	YP_009094330	Non-RABV-PI	0.54 µg/mL
ARAV	AAP86775	Non-RABV-PI	31.62 µg/mL
MOKV	AAB26296	Non-RABV-PII	14.74 µg/mL
LBV	MH643893	Non-RABV-PII	4.84 µg/mL
SHIBV	GU170201	Non-RABV-PII	3.18 µg/mL ^#^

^#^ The maximum infection inhibition ratio of SYN023 against the SHIBV variant was 56.27%.

**Table 2:** Comparative neutralization activity of SYN023 versus other anti-rabies agents against the MOKV lyssavirus variant.

Neutralization Against MOKV (EC_50_)				
SYN023	Rabishield	Ormutivimab	GR1801	HRIG
14.74 µg/mL	>1000 µg/mL *	>1000 µg/mL *	>1000 µg/mL *	0.229 IU/mL

* No neutralizing activity was detected at the concentration.

## Data Availability

The data of this study are available upon reasonable request from the authors.

## Supplementary Materials

The following supporting information can be downloaded at: https://www.mdpi.com/article/10.3390/vaccines14100862/s1, Table S1: Neutralization activity of SYN023 against authentic and pseudotyped lyssaviruses; Table S2: Observational summary of NYC rabies virus titration in naïve dogs; Table S3: Observational summary and diagnostic test results (DFA and RVNA) evaluating SYN023 efficacy in canine PEP.

## Abbreviations

The following abbreviations are used in this manuscript:

RABV	Rabies virus
NYC	New York City rabies virus strain
PEP	Post-exposure prophylaxis
EBLV1	European bat lyssavirus 1
EBLV2	European bat lyssavirus 2
ABLV	Australian bat lyssavirus
BBLV	Bokeloh bat lyssavirus
TWBLV1	Taiwan bat lyssavirus 1
TWBLV2	Taiwan bat lyssavirus 2
KBLV	Kotalahti bat lyssavirus
DBLV	Divača bat lyssavirus
GBLV	Gannoruwa bat lyssavirus
DUVV	Duvenhage virus
KHUV	Khujand virus
ARAV	Aravan virus
MOKV	Mokola virus
LBV	Lagos bat virus
SHIBV	Shimoni bat virus
WHO	World Health Organization
CFIA	Canadian Food Inspection Agency
CDC	Centers for Disease Control and Prevention
RVNA	Rabies virus neutralizing antibody
RFFIT	Rapid fluorescent focus inhibition test
DFA	Direct fluorescent antibody test
