# Supplementary material for: SYN023, a Humanized Monoclonal Antibody Cocktail, Broadly Neutralizes Genetically Diverse Lyssaviruses and Validates Epitope Prediction Accuracy
Source: Vaccines (Basel). 2026 Sep 29;14(10):862. doi: 10.3390/vaccines14100862 (PMC13709800; doi:10.3390/vaccines14100862)
Supplement: Supplementary file 1 [file vaccines-14-00862-s001.zip › vaccines-4567969-supplementary.pdf]

Table S1. Neutralization activity of SYN023 against authentic and pseudotyped lyssaviruses

Lyssavirus variant	Taxonomy	Region	CTB012 epitope				CTB011 epitope			Neutralization of SYN23 EC50 (μg/ml)	
			286	289	290	291	355	361	389	Authentic virus	Pseudovirus
CVS-11	RABV	Laboratory	E	H	L	V	N	K	H	3.79E-03	5.51E-03
QH2	RABV	Qinghai, China	E	H	L	V	N	K	H	7.43E-03	1.65E-03
YN3	RABV	Yunnan, China	E	H	L	V	N	K	H	9.71E-03	6.11E-03
SHB	RABV	Saskatch-ewan, Canada	E	H	L	V	N	K	N	3.08E-01	8.97E-02
EBLV2	EBLV2	Finland	E	H	L	I	T	K	N	2.08E+01	8.22E+00
CUDOG	RABV	Cuba	E	H	L	V	N	K	H	6.17E-02	5.67E-04
SLDOG	RABV	Sri Lanka	E	H	L	V	N	K	H	5.49E-02	7.66E-04
ARCNu	RABV	Nunavut, Canada	E	H	L	V	N	K	H	4.39E-02	1.52E-03
ARC#3	RABV	Eastern Ontario, Canada	E	H	L	V	N	K	H	4.39E-02	1.39E-03
SC16	RABV	Sichuan, China	E	H	L	V	N	K	H	8.33E-03	8.07E-04
LY	RABV	Ningxia, China	E	H	L	V	N	K	H	5.14E-03	2.50E-03
NM3	RABV	Inner Mongolia, China	E	H	L	V	N	K	H	1.05E-02	2.23E-03
BBLV	BBLV	France	E	H	L	I	T	K	N	1.19E+01	9.58E-01
ERA	RABV	Laboratory	E	H	L	V	N	K	N	5.56E-02	3.72E-03
Parastrellus hesperus	RABV	Arizona, USA	E	H	L	V	N	K	N	4.70E-03	1.79E-03
Molossus molossus	RABV	Argentina	E	H	L	V	N	K	N	7.72E-02	5.25E-03
AFRM	RABV	South Africa	E	H	L	V	D	K	H	3.91E-02	1.87E-03
NCSK	RABV	USA	E	H	L	V	N	K	H	3.91E-03	6.39E-03
Lasiurus borealis	RABV	USA	E	H	L	V	N	K	N	1.60E-03	2.86E-03
Lasiurus cinereus	RABV	USA	E	H	L	V	N	K	N	8.47E-02	4.16E-01
Antrozous pallidus	RABV	California, USA	E	H	L	V	N	K	H	1.09E-03	1.64E-03
USScSK	RABV	Texas, USA	E	H	L	V	N	K	H	1.10E-02	1.37E-03

Table S2. Observational summary of NYC rabies virus titration in naïve dogs

Group	Animal ID	Euthanize/ Deceased	Date of Euthanasia/	Days post inoculation	Observations
Group 1 NYC street virus 1:10	CHBCGL	Euthanized	2016.05.12	7	Constantly biting left hind leg. Ataxia
	CHBCGN	Euthanized	2016.05.12	7	Lethargic, favoring left hind leg
	CHBCJS	Euthanized	2016.05.12	7	Spinning in circles, biting left hind leg
Group 2 NYC street virus 1:100	CHBCGM	Euthanized	2016.05.14	10	Hind limb paresis; animal very unsteady and unstable; hides in back corner of cage
	CHBCJP	Euthanized	2016.05.15	11	Did not eat, unstable when walking. L hind leg shakes without much control
	CHBCKK	Euthanized	2016.05.14	10	Hind limb paralysis, L leg
Group 3 NYC street virus 1:1000	CHBCHG	Euthanized	2016.05.13	9	Biting left hind foot/leg. Ataxia
	CHBCHY	Euthanized	2016.06.28	54	Noted to be bright, alert, and responsive over study duration, euthanized at study Day 54
	CHBCIA	Euthanized	2016.05.14	10	Hind end shaky, decreased movement; animal keeps hind end hunched down; not as

Table S3. Observational summary and diagnostic test results (DFA and RVNA) evaluating SYN023 efficacy in canine PEP.

Group	Animal ID	Euthanized / Deceased	Days Post Inoculation	Observations	DFA result	RVNA (IU/mL)
Group 1<br>Challenge:<br>NYC street virus at 1:100 dilution<br><br>Treatment:<br>SYN023 at 0.5 mg/kg	CHDCHY	Euthanized	90	Noted to be bright, alert, and responsive over study duration, euthanized at study termination	Negative	0.5
	CHDCIU	Euthanized	90	Noted to be bright, alert, and responsive over study duration, euthanized at study termination	Negative	0.9
	CHDCJX	Euthanized	90	Noted to be bright, alert, and responsive over study duration, euthanized at study termination	Negative	1.2
	CHDCKE	Euthanized	90	Noted to be bright, alert, and responsive over study duration, euthanized at study termination	Negative	0.5
	CHDCKH	Euthanized	90	Noted to be bright, alert, and responsive over study duration, euthanized at study termination	Negative	0.3
	CHDCKI	Euthanized	90	Noted to be bright, alert, and responsive over study duration, euthanized at study termination	Negative	0.3
Group 2<br>Challenge:<br>NYC street virus at 1:100 dilution<br><br>Treatment:<br>SYN023 at 0.3 mg/kg	CHDCGE	Euthanized	90	Noted to be bright, alert, and responsive over study duration, euthanized at study termination	Negative	0.5
	CHDCGF	Euthanized	90	Noted to be bright, alert, and responsive over study duration, euthanized at study termination	Negative	0.5
	CHDCJA	Euthanized	11	Lack of hind limb coordination, stiffness in hind legs, decreased appetite, biting hind limbs	Positive	2.4
	CHDCKC	Euthanized	90	Noted to be bright, alert, and responsive over study duration, euthanized at study termination	Negative	0.3
	CHDCKJ	Euthanized	90	Noted to be bright, alert, and responsive over study duration, euthanized at study termination	Negative	1.2
	CHDCKL	Euthanized	90	Noted to be bright, alert, and responsive over study duration, euthanized at study termination	Negative	0.9
Group 3<br>Challenge:<br>NYC street virus at 1:100 dilution<br><br>Treatment:<br>SYN023 at 0.1 mg/kg	CHDCHC	Euthanized	90	Noted to be bright, alert, and responsive over study duration, euthanized at study termination	Negative	0.5
	CHDCJJ	Euthanized	90	Noted to be bright, alert, and responsive over study duration, euthanized at study termination	Negative	0.5
	CHDCJW	Euthanized	90	Noted to be bright, alert, and responsive over study duration, euthanized at study termination	Negative	2.4
	CHDCKA	Euthanized	90	Noted to be bright, alert, and responsive over study duration, euthanized at study termination	Negative	0.3
	CHDCKF	Euthanized	9	Only ate 1/2 of food. Excessive licking of genital area and left rear leg. Favoring left rear leg. Beginning to become shaky when trying to walk	Positive	1.2
	CHDCKK	Euthanized	90	Noted to be bright, alert, and responsive over study duration, euthanized at study termination	Negative	0.9
Group 4<br>Challenge:<br>NYC street virus at 1:100 dilution<br><br>Treatment:<br>PBS	CHDCHR	Euthanized	10	Hind leg paresis, unwilling to move	Positive	3.4
	CHDCHT	Euthanized	9	Tries to move, but very shaky. Favoring left hind leg	Positive	3.7
	CHDCIJ	Euthanized	10	Appears shaky. Favoring left hind leg	Positive	3.4
	CHDCJK	Euthanized	8	Excessive licking of genital area, favoring/shaking left hind leg	Positive	3.4
	CHDCKD	Euthanized	8	Some excessive licking of genital area. Favoring/walking abnormally on occasion left hind leg. Occasionally looks back at injection site on left leg and attempts to bite it	Positive	3.4
	CHDCKG	Euthanized	10	Excessive licking of genital area and left hind leg	Positive	3.4
